# Gastrointestinal stromal tumor: 15-years’ experience in a single center

**DOI:** 10.1186/1471-2482-14-93

**Published:** 2014-11-18

**Authors:** Ming Wang, Jia Xu, Yun Zhang, Lin Tu, Wei-Qing Qiu, Chao-Jie Wang, Yan-Ying Shen, Qiang Liu, Hui Cao

**Affiliations:** Department of General Surgery, Ren Ji Hospital, School of Medicine, Shanghai Jiao Tong University, Floor 11, Building 7, NO. 1630, Dongfang Road, Shanghai, 200127 China; Department of Pathology, Ren Ji Hospital, School of Medicine, Shanghai Jiao Tong University, Shanghai, China

**Keywords:** Gastrointestinal stromal tumor, Survival, Imatinib

## Abstract

**Background:**

Gastrointestinal stromal tumor (GIST) is known for its wide variability in biological behaviors and it is difficult to predict its malignant potential. The aim of this study is to explore the characteristics and prognostic factors of GIST.

**Methods:**

Clinical and pathological data of 497 GIST patients in our center between 1997 and 2012 were reviewed.

**Results:**

Patients were categorized into very low-, low-, intermediate- and high-risk groups according to modified National Institutes of Health (NIH) consensus classification system. Among the 401 patients untreated with imatinib mesylate (IM), 5-year overall survival (OS) in very low-, low-, intermediate- and high-risk groups was 100%, 100%, 89.6% and 65.9%; and 5-year relapse-free survival (RFS) was 100%, 98.1%, 90.9% and 44.5%, respectively. Univariate analysis revealed that sex, tumor size, mitotic rate, risk grade, CD34 expression, and adjacent involvement were predictors of OS or RFS. COX hazard proportional model (Forward LR) showed that large tumor size, high mitotic rate, and high risk grade were independent risk factors to OS, whereas high mitotic rate, high risk grade and adjacent organ involvement were independent risk factors to RFS. The intermediate-high risk patients who received IM adjuvant therapy (n = 87) had better 5-year OS and RFS than those who did not (n = 188) (94.9% vs. 72.1; 82.3% vs. 56.3%, respectively). Similarly, advanced GIST patients underwent IM therapy (n = 45) had better 3-year OS and 1-year progression-free survival (PFS) than those who didn’t (n = 42) (75.6% vs. 6.8%; 87.6% vs. 12.4%, respectively).

**Conclusions:**

Very low- and low-risk GISTs can be treated with surgery alone. Large tumor size, high mitotic rate, high risk grade, and adjacent organ involvement contribute to the poor outcome. IM therapy significantly improves the survival of intermediate-high risk or advanced GIST patients.

**Electronic supplementary material:**

The online version of this article (doi:10.1186/1471-2482-14-93) contains supplementary material, which is available to authorized users.

## Background

Gastrointestinal stromal tumor (GIST) is the most common mesenchymal neoplasm in the gastrointestinal (GI) tract [1]. Mazur and Clark [2] first introduced the concept of “stromal tumor” in 1983. Advance in pathology, immunohistochemistry and molecular biology in recent years has greatly improved the diagnosis of GIST. It is now considered that GISTs arise from interstitial Cajal cells (ICCs), expressing CD117 (product of c-kit proto-oncogene), and harboring c-kit or platelet-derived growth factor receptor alpha (PDGFRA) gain-of-function mutation [3–5].

GIST is known for its wide variability in biological behaviors and it is difficult to predict its malignant potential [6, 7]. Tumor size, mitotic rate and tumor site are considered as the most important prognostic parameters for patients after surgery [8]. However, neither small size nor low mitotic rate could exclude malignant potential [9]. On the other hand, some enormous tumor with high mitotic rate could also achieve long-term survival, even without adjuvant therapy [10]. The post-operation outcome of GIST is highly variable, with 5-year survival rate ranging from 48% to 80% [11, 12]. The variability is mainly due to the introduction of a tyrosine kinases inhibitor (TKI), imatinib mesylate, which was used in metastatic/recurrent GISTs since 2000 and had been proved as an adjuvant therapy several years ago [13, 14].

The purpose of this study is to share our latest 15 years of experience and to explore the prognostic factors of GISTs.

## Methods

The clinicopathological and follow-up data of 497 operable GIST patients admitted to Department of General Surgery, Ren Ji Hospital, School of Medicine, Shanghai Jiao Tong University between 1997 and 2012 were reviewed. Each diagnosis of “GIST” was confirmed by postoperative histopathology and immunohistochemistry assay (IHCA). The results of histopathological features and IHCA findings of every case were reviewed by 2 experienced pathologists. Those diagnosed as “gastrointestinal stromal mesenchymal tumor” prior to 2000 were re-examined by IHCA to confirm the diagnosis of GIST. The tumors were categorized into very low, low, intermediate and high risk groups according to the modified NIH risk classification criteria [7] (Table 1). Only the cases with complete medical records and pathological data were involved in present study. The following parameters were reviewed and analyzed: age, sex, clinical presentation, surgical detail, tumor site, tumor size, mitotic rate, IHCA (CD117, CD34, vimentin, smooth muscle actin (SMA), S-100, Discovered On GIST 1 (DOG1)), TKI therapy and outcome. Survival outcome in terms of overall survival (OS), relapse-free survival (RFS), and progression-free survival (PFS) were assessed. OS was defined as the period from surgery to the last follow-up or death. RFS was defined as the period from surgery to the time of clinical or radiological evidence of disease relapse. PFS in patients who had metastatic or recurrent disease was defined as the period from the time when relapse was diagnosed to clinical or radiological evidence of progression or death.

**Table 1:** **Risk classification of GISTs**

Risk classification	Tumor size (cm)	Mitotic rate per 50 HPF	Tumor site
Very low risk	<2	<=5	Any
Low risk	2.1-5.0	<=5	Any
	2.1-5.0	>5	Gastric
Intermediate risk	<5	6-10	Any
	5.1-10	<=5	Gastric
High risk	Any	Any	Tumor rupture
	>10	Any	Any
	Any	>10	Any
	>5	>5	Any
	2.1-5.0	>5	Non gastric
	5.1-10.0	<=5	Non gastric

HPF = high power field.

All patients provided written informed consent for their information to be stored in the hospital database, and we obtained separate consent for use of research. Study approval was obtained from independent ethics committees from Ren Ji Hospital, School of Medicine, Shanghai Jiao Tong University. The study was undertaken in accordance with the ethical standards of the World Medical Association Declaration of Helsinki.

χ^2^ test and Fisher’s exact test were performed to analyze qualitative parameters and Kaplan-Meier method with log rank test was used for postoperative survival analysis. Independent factors were identified in multivariate analysis by COX proportional hazard analysis with forward selection at P < 0.05. Odds ratios (ORs) and 95% confidence intervals (CIs) were determined using unconditional multiple logistic regression models. Two-sided P values of 0.05 or less were considered to indicate statistical significance.

## Results

**Figure 1:**
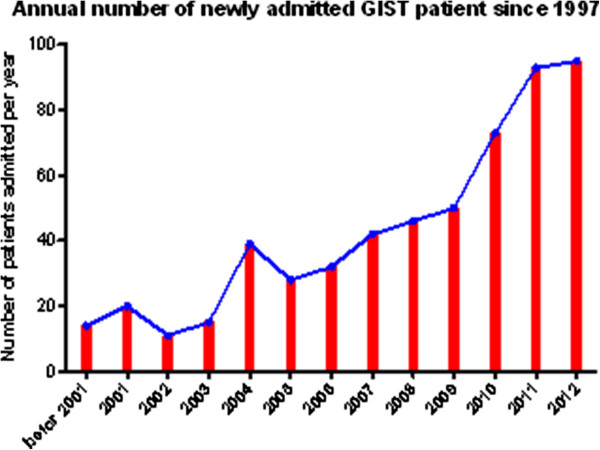
**Annual number of newly admitted GIST patient since 1997.**

The incidence of GIST ranges from 11 to 15 per million per year [15–18]. Growing evidence indicates the incidence is considerably underestimated [19, 20]. The number of GIST patients admitted to our center is on the rise. In the past two year, it has approached 100 cases a year (Figure 1).

### Clinical and pathological characteristics

Total 497 GIST patients were involved in present study, with a median age of 60 years (range 23–90) and 55.9% was male. Stomach and small bowel were the most common sites of primary disease (59.0% and 22.5%, respectively). The most common clinical presentation was abdominal discomfort, followed by GI bleeding. Distribution of risk groups: 8.0%, very low; 36.4%, low; 15.7%, intermediate; and 39.8%, high risk. IM adjuvant therapy was given to 96 of the patients to prevent disease relapse. Recurrence or metastases were observed in 89 patients during the follow-up period. Among which, IM was used to control disease in 46 patients.

Of all the cases, 87.3% was CD117 (+); 80.3%, CD34 (+); 23.6%, SMA (+); and 21.5%, S-100 (+). DOG1 was a newly developed IHCA marker, which was positive in 139 out of 149 cases (93.3%). The diagnosis of GIST in patients presented as both CD117 and DOG1 negative was confirmed by detection of mutation in c-kit/PDGFRA gene. Their clinical and pathological characteristics are listed in Table 2.

**Table 2:** **Clinical and pathological characteristics of 497 GIST patients**

**Age (years)**	
Median	60
Range	23-90
**Sex, n (%)**	
Male	278 (55.9)
Female	219 (44.1)
**Primary site of tumor, n (%)**	
Stomach	293 (59.0)
Duodenum	31 (6.2)
Small bowel	112 (22.5)
Large bowel	4 (0.8)
Rectum	21 (4.2)
Esophagus	3 (0.6)
Other (omentum, mesenterium and retroperitoneum )	33 (6.6)
**Clinical manifestation, n (%)**	
Abdominal discomfort	184 (37.0)
GI bleeding	142 (28.6)
Diagnosed at physical examination	81 (16.3)
Abdominal mass	14 (2.8)
Other (fever, fatigue, appetite and explored at surgery for other diseases)	76 (15.3)
**IM therapy**	
As adjuvant therapy for primary disease	96
As therapy for advanced disease (recurrent, metastatic, unresectable, or incomplete resected)	46
**Immunohistochemistry, n (%)**	
CD117	434 (87.3)
CD34	399 (80.3)
SMA	119 (23.9)
S-100	107 (21.5)
DOG1	139 (93.3)*

*DOG1 was examined in 165 cases.

Lymph node metastasis was detected in 5 out of 497 cases (1.01%); clinical and pathological characteristics of these 5 cases were described in Table 3.

**Table 3:** **Clinical and pathological characteristics of 5 GIST patients with lymph node metastasis**

Case	Sex	Age	Primary site	Tumor size	Mitotic rate per 50HPF	MLN/TLN	Mutation	IM therapy	Outcome
1	M	60	S	8	23	2/4	c-kit exon9	Yes	Died of disease progression at 33 months after surgery
2	M	58	G	5	12	1/2	not available	No	DFS at 101 months after surgery
3	F	59	G	5.5	<5	4/4	c-kit exon11	Yes	DFS at 45 months after surgery
4	F	70	G	9	8	1/9	c-kit exon11	Yes	DFS at 25 months after surgery
5	M	31	D	18	<5	2/2	c-kit exon11	Yes	Survival with residual disease at 3 months after surgery

S = small intestine; G = stomach; D = duodenum MLN = metastatic lymph nodes; TLN = total examined lymph nodes; DFS = Disease-free survival.

### Survival analysis on patients without IM adjuvant therapy

Given the fact that imatinib is an effective drug on GIST, the first survival analysis was based on the population of patients who were not given IM adjuvant therapy. Therefore, 401 patients with operable GIST were enrolled in the cohort, with a median duration of 50 months (range, 7–187 months). Recurrence or metastasis occurred in 79 patients (19.7%). The abdominopelvic cavity was the most common site of metastases (51 cases), followed by liver (22 cases), lung (3 cases), vertebral column (1 case), umbilicus (1 case), and fossa axillaris (1 case). Forty-five patients died of GIST progression, and 4 died of other diseases. The 1-, 3-, 5-year OS of 401 GISTs was 97.7%, 92.6% and 84.8%, respectively; The 1-, 3-, 5-year RFS was 93.2%, 82.1% and 77.4%, respectively.

**Figure 2:**
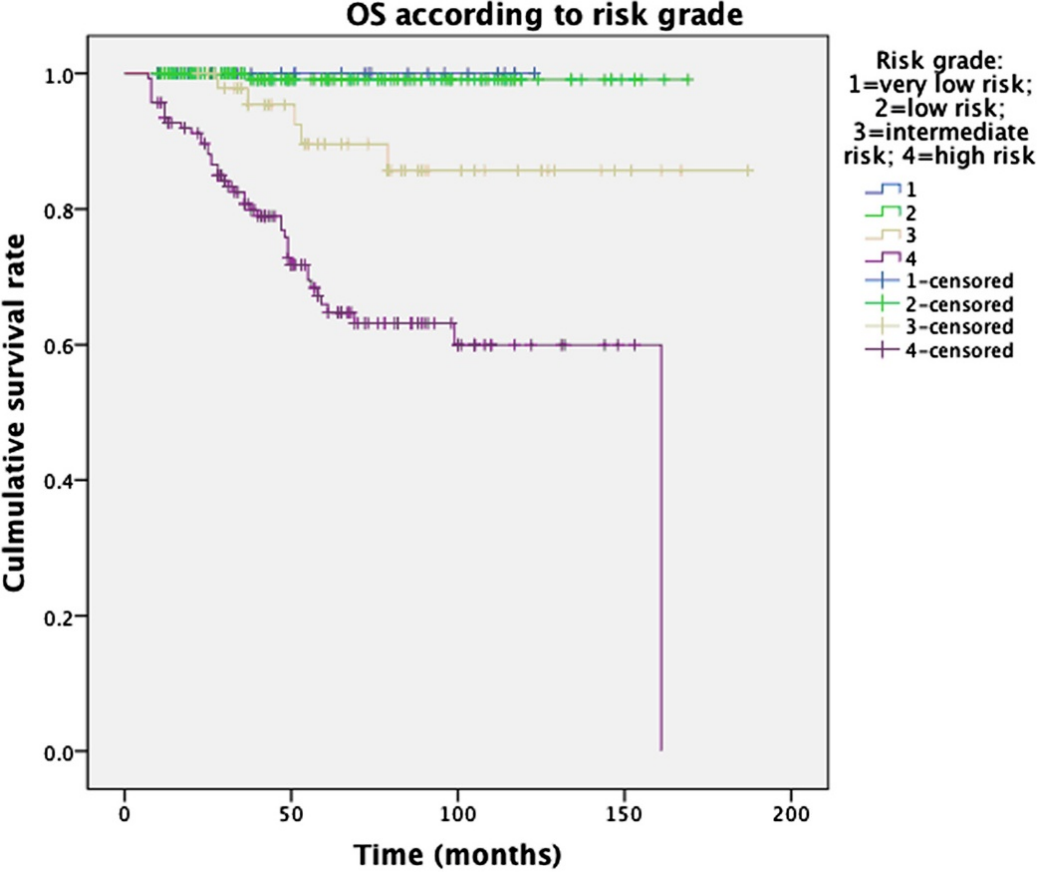
**Overall survival in 401 GIST patients according to risk class.**

The 1-, 3-, 5-year OS according to risk grade was: 100%, 100%, 100% (very low risk); 100%, 100%, 100% (low risk); 100%, 97.8%, 89.6% (intermediated risk); 93.5%, 80.8%, 65.9% (high risk), respectively (Figure 2).

**Figure 3:**
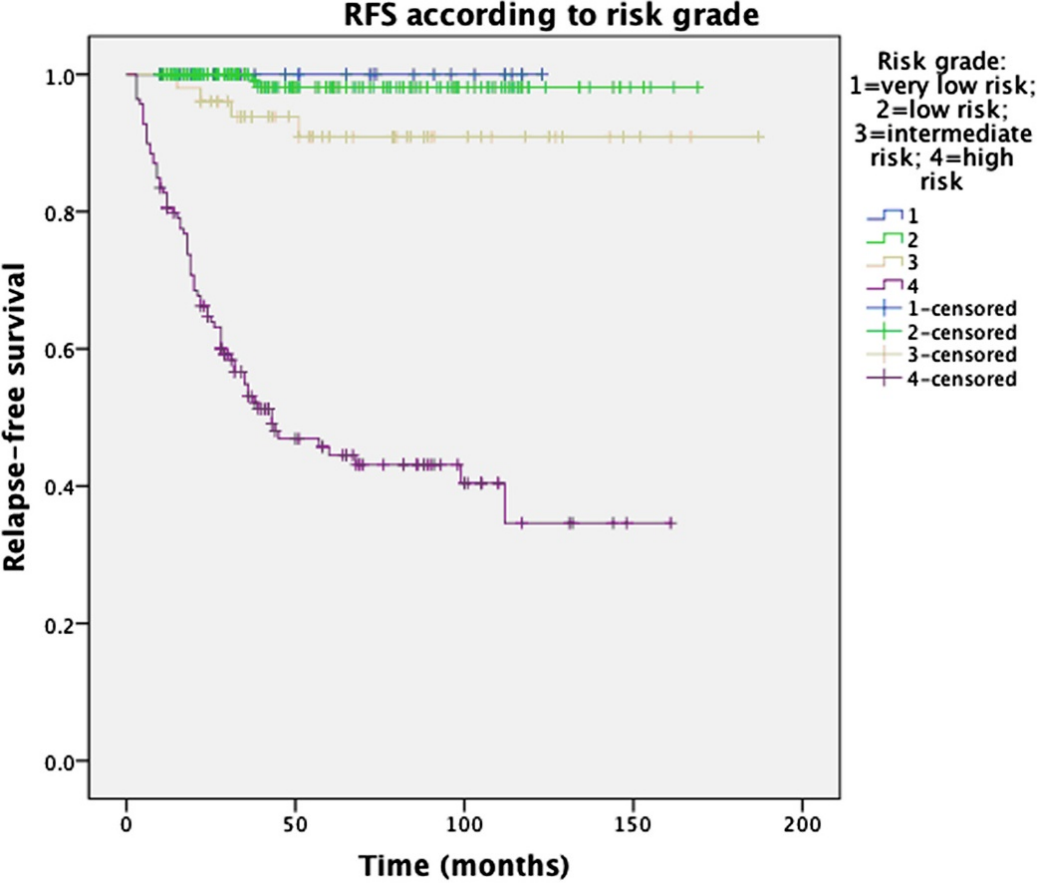
**Relapse-free survival in 401 GIST patients according to risk class.**

The 1-, 3-, 5-year RFS according to risk group was: 100%, 100%, 100% (very low risk); 100%, 100%, 98.1% (low risk); 100%, 93.8%, 90.9% (intermediated risk); 80.6%, 53.1%, 44.5% (high risk), respectively (Figure 3).

Univariate analysis revealed that male gender, non-gastric origin, larger tumor size, higher mitotic rate, higher risk grade, CD34 negative expression, and adjacent organ involvement contributed to poorer outcome (lower OS and RFS), whereas age and expression of CD117, SMA, and S-100 were not associated with prognosis (see Table 4 and Additional files 1 and 2).

**Table 4:** **Univariate analysis of OS and RFS in 401 GIST patients**

Clinicopathological feature	Group	N	OS			RFS		
			5-year OS (%)	χ ^2^	P-value	5-year RFS (%)	χ ^2^	P-value
Gender	Male	221	80.2	6.590	0.010	71.6	8.914	0.003
	Female	180	90.6			84.4		
Age	<60	197	85.7	1.573	0.210	77.4	0.011	0.917
	≥60	204	84.0			77.4		
Tumor site	Gastric	241	86.5	1.969	0.161	86.1	18.876	<0.001
	Non-gastric	160	82.5			65.3		
Tumor size	≤2 cm	51	100	110.281	<0.001	100	146.144	<0.001
	2.1-5 cm	178	98.0			94.6		
	5.1-10 cm	109	80.9			66.8		
	>10 cm	63	49.2			33.5		
Mitotic rate	<5/50 HPF	296	95.9	83.348	<0.001	91.2	152.472	<0.001
	5-10/50 HPF	44	68.0			55.3		
	>10/50 HPF	61	54.2			33.7		
Risk class	Very low	39	100	66.044	<0.001	100	154.234	<0.001
	Low	172	99.1			98.1		
	Intermediate	51	89.6			90.9		
	High	139	65.9			44.5		
CD117 expression	Positive	350	83.4	3.315	0.069	76.6	1.401	0.237
	Negetive	51	95.1			82.6		
CD34 expression	Positive	322	87.2	7.564	0.006	80.6	10.777	0.001
	Negative	79	75.4			64.3		
SMA expression	Positive	101	90.6	1.559	0.212	83.2	2.246	0.134
	Negative	300	83.2			75.6		
S-100 expression	Positive	84	88.2	1.377	0.241	74.0	0.529	0.467
	Negative	317	83.7			78.5		
Adjacent involvement	Without	336	92.1	66.176	<0.001	87.2	147.885	<0.001
	With	65	53.0			30.8		

Multivariate analysis by Cox proportional hazards regression (Forward LR) model indicated that tumor size, mitotic rate, and risk grade were independent risk factors to OS for GISTs, and that mitotic rate, risk grade, and adjacent involvement were independent risk factors to RFS (Tables 5 and 6).

**Table 5:** **Multivariate COX regression analysis of OS in 401 GIST patients**

Covariate	χ ^2^	P-value	Hazard ratio	95% CI
Tumor size (> = 10 cm vs. <10 cm)	13.224	<0.001	3.293	1.732-6.261
Mitotic rate (> = 5/50 HPF vs. <5/50 HPF)	10.619	0.001	3.841	1.710-8.628
Risk grade (high risk vs. non-high risk)	4.956	0.026	3.440	1.159-10.207

**Table 6:** **Multivariate COX regression analysis of RFS in 401 GIST patients**

Covariate	χ ^2^	P-value	Hazard ratio	95% CI
Adjacent involvement (with vs. without)	11.841	0.001	2.295	1.430-3.683
Mitotic rate (> = 5/50 HPF vs. <5/50 HPF)	8.895	0.003	2.406	1.351-4.284
Risk grade (high risk vs. non-high risk)	26.129	<0.001	11.794	4.579-30.379

### Survival analysis of patients received IM therapy

**Figure 4:**
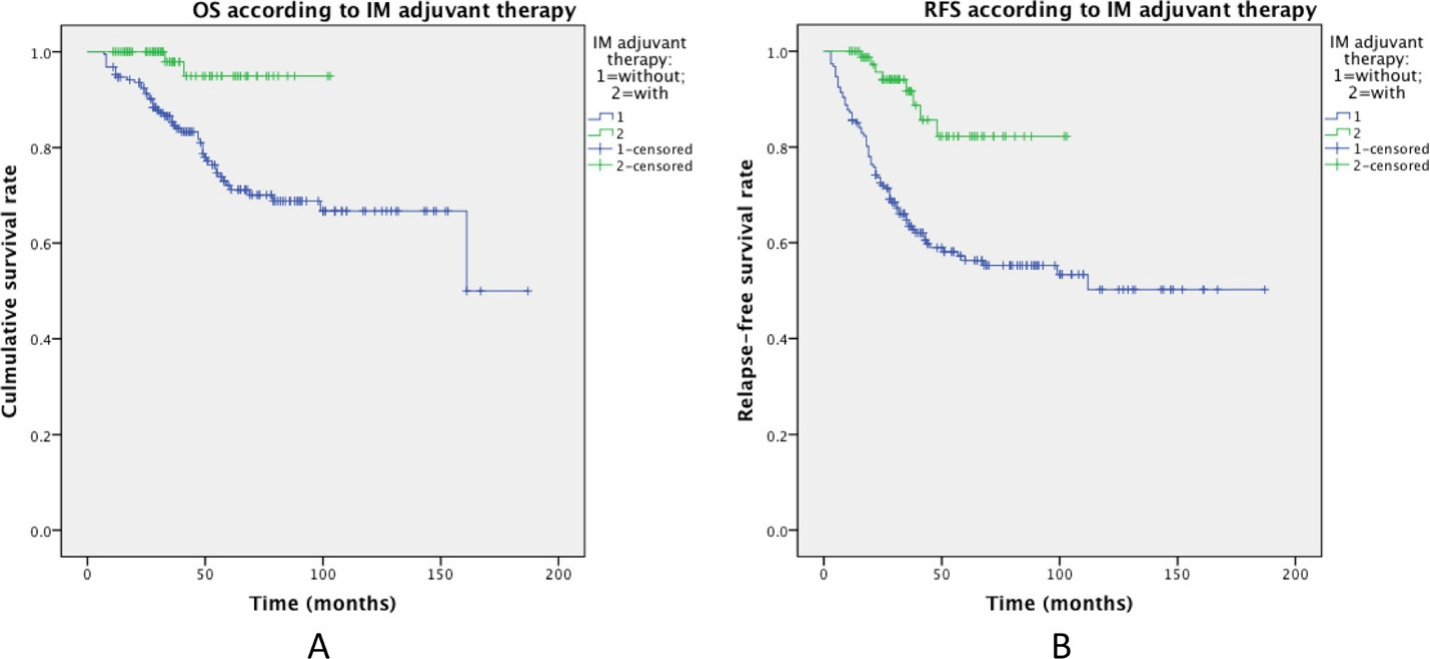
**Effect of treatment with or without postoperative IM adjuvant therapy on OS and RFS in 275 intermediate-high risk GIST patients (A: overall survival; B: relapse-free survival).**

From 2007 to 2012, 87 patients with intermediate-high risk GIST received IM adjuvant therapy after radical resection (Adjuvant group). Compared with those patients who were with same risk GIST (intermediate-high risk) while were not given IM adjuvant therapy (Non-adjuvant group, n = 188), adjuvant group had better 5-year RFS (82.3% vs. 56.3%, P < 0.001) and 5-year OS (94.9% vs. 72.1%, P = 0.001) (Figure 4). In addition, there was no statistical difference in other clinicopathological features (sex, age, tumor site, tumor size, mitotic rate, risk grade, etc.) between the two groups (see Additional file 3), indicating that these features had no impact on the effect of IM.

**Figure 5:**
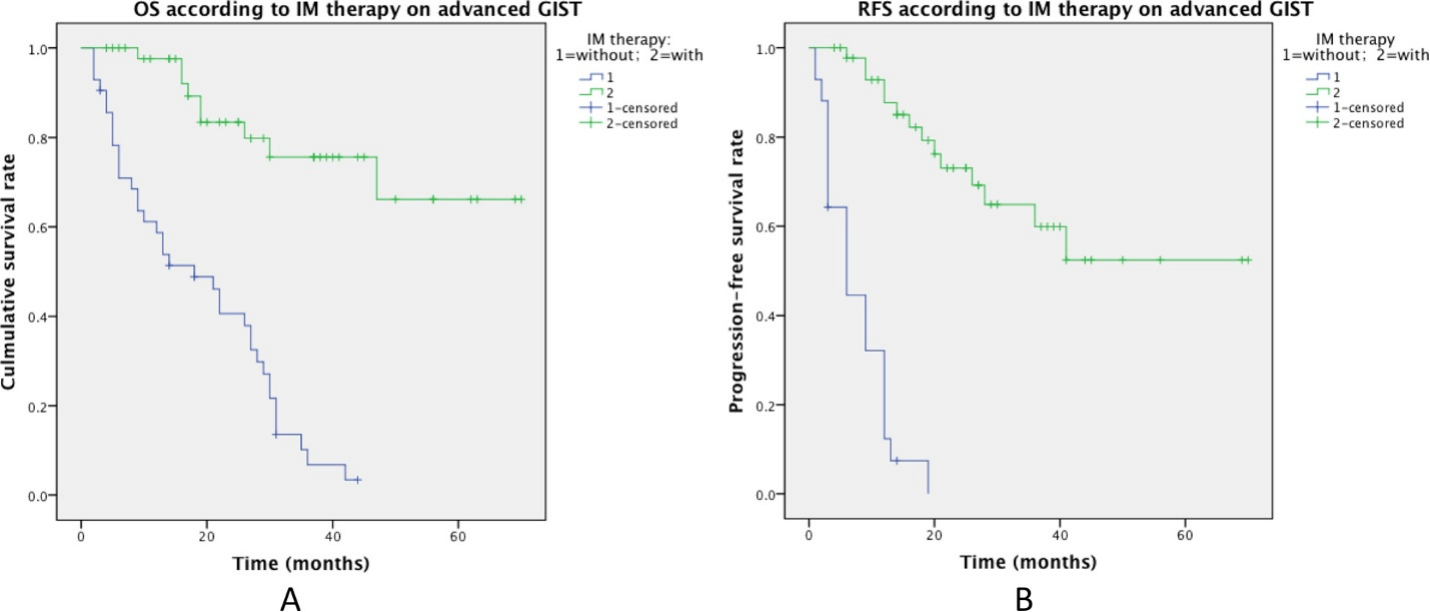
**Effect of treatment with or without post-operation IM therapy on OS and PFS in 87 advanced GIST cases (A: overall survival; B: progression-free survival).**

In the cohort, 87 patients developed recurrence of metastasis after surgery for the primary disease. Among them, c-kit/PDGFRA mutation status was screened in 39 patients. Their mutational characteristics were demonstrated in our previous report [21]. Mutations in c-kit exon 11, c-kit exon 9, and PDGFRA exon 18 were identified in 29, 4, and 1 patients, respectively. And the rest 5 GISTs showed c-kit and PDGFRA wild type. Among all the 87 advanced GIST patients, 45 (including 33 c-kit mutant GISTs, 5 wild-type GISTs, and 7 GISTs with unknown mutation type) were treated with IM, and the other 42 didn’t undergo any TKI therapy (10 due to personal reasons and the rest were cases prior to 2005). There was significant difference in outcome between the two groups: patients underwent postoperative IM treatment had better 1-, 3-year OS than those untreated with IM (97.6% and 75.6% vs. 58.7% and 6.8%, respectively, P < 0.001). IM therapy also improved 1-year progression-free survival (PFS) of these patients (87.6% vs. 12.4%, P < 0.001) (Figure 5).

## Discussion

Although the incidence of GISTs is rising in the oriental population, available document on this area is still limited, especially studies with large sample size in a single center. This study reviewed the clinical and pathological features of 497 GIST cases in Shanghai Ren Ji Hospital to explore the prognostic factors of the disease.

GISTs represent 80% of mesenchymal tumor of the digestive tract and constitute 5% of all sarcoma [22]. It had been reported that the annual occurrences of GIST were 11–15 per million people [15–18]. However, growing evidences have proved that the incidence of GISTs is seriously underestimated. Learn from the studies of Abraham et al. [23] and Agaimy et al. [24], we can draw a conclusion that sub-centimeter GISTs (smaller than 1 cm) are common lesions in stomach. Our epidemiologic data show the number of newly diagnosed GISTs is on the fast rise (Figure 1), probably due to the increasing awareness of the disease in clinicians.

Our data indicate that GIST occurrences culminate among people in their 50s and 60s. The youngest GIST patient is a 23-year-old female, who suffered from giant retroperitoneal GIST and died of recurrent disease 32 months after surgery. The oldest patient is a 90-year-old male with intermediate-risk gastric GIST and he was relapse-free at last follow-up, six months after surgery. Although in most published documents there is no clear sex predilection [3, 25, 26], some studies revealed that there was a slight male predominance [27–29]. Our data agree with the latter.

GISTs have no specific symptom, increasing the difficulty in early diagnosis and treatment. In our data, consistent with the literature, the most frequent complaint is abdominal discomfort, which may or may not be accompanied by GI bleeding [30, 31].

GIST may arise anywhere in the GI tract and also in extragastrointestinal locations (extragastrointestinal stromal tumor, EGIST), including omentum, mesenterium, and retroperitoneum [32]. According to our data, the most common GI location of primary disease was stomach (59.0%), followed by small bowel (22.5%), duodenum (6.2%), rectum (4.2%), large bowel (0.8%), esophagus (0.6%). EGISTs were found in 6.6% of cases.

Typical GISTs are characterized by positive immunohistochemical (IHC) staining of KIT (CD117), a trans-membrane receptor tyrosine kinase. More recently the antigen DOG1 has been incorporated in the IHC panel when CD117 was negative [33]. Our data confirmed the high specificity and sensitivity of this marker: DOG1 expression was seen in 139 of 149 GISTs, including 15 CD117 negative ones.

Except for some sporadic studies [34], lymph node metastasis is reported to be extremely rare in GIST, with incidence ranging from 0 ~ 5% [11, 35–37]. Although lymph node metastasis (LNM) is usually considered as a morphological feature associated with malignancy and poor prognosis [38, 39], our data do not support this opinion. Three out of the 5 patients with LNM in this study achieved longer than 2 years’ DFS; one of them, though untreated with IM, was still disease-free at the latest follow-up, over 8 years after surgery. This aroused the controversy over the exact impact of LNM on GIST outcome. Further studies with larger sample size are required to solve this puzzle. Nonetheless, lymph node dissection should be considered in case of suspected or confirmed LNM.

The distribution of very low-, low-, intermediate-, high-risk groups was 8.0%, 36.4%, 15.7%, and 39.8%, respectively. Compared to most published literature [28, 40, 41], the proportion of very low and low risk GIST was much higher. One reason might be the improved screening system and early surgery. In addition, clinical study on minimal invasive procedure (laparoscopic/laparoscopy-endoscopy cooperative surgeries) for GIST is being conducted in our center, and offers the opportunity of early operation and further elevated the proportion. Thanks to the popularity of endoscopy, more and more GISTs can be determined at small size. In most cases, having the advantages of small incision and fast recovery, minimal invasive surgery is preferred to traditional open operation.

In our study, the outcome (both OS and RFS) of IM-naive GIST patients was better than that in most published literature [12, 42, 43] for the same reasons mentioned above (higher proportion of low risk GIST). Nonetheless, the high-risk group still had unsatisfactory results (5-year OS 65.9%, 5-year RFS 44.5%, respectively). However, very low- and low-risk GISTs in present study had rather better prognosis: no relapse was found in very low risk group; only one case, a rectal tumor 3.5cm in diameter with mitotic rate of <5/50HPF, occurred recurrence in low-risk group.

Prediction of biological behavior of a GIST is essential for selection of candidates for adjuvant therapy as well as determination of the frequency and intensity of postoperative surveillance. However, accurate prediction is often a difficult job. It has been widely accepted that tumor size, mitotic rate, and anatomic site are the most important factors influencing the prognosis of GISTs [8]. These factors form the basis for consensus risk classification. Our study reveals that risk grade and mitotic rate were independent prognostic factors of both OS and RFS, while tumor size and adjacent organ involvement was independent predictor of OS and RFS, respectively. Mitotic rate was described as a vital indicator for GIST staging and consequential choice of surgical and target therapeutic approach [44, 45], its value in prognosis prediction was confirmed again in our study. It’s worth mentioning that there is a difficulty in reproducibility among examiners when determining the mitotic rate [44]. Therefore, all specimens should be examined by specialized experts to decrease the deviation.

In present study, males had lower survival rate than females (5-yaer OS, 80.2% vs. 90.6%, P = 0.010; 5-year RFS, 71.6% vs. 84.4%, P = 0.003). This finding was in consistent with other retrospective studies [46–48]. However, no relationship between sex and survival was found in the multivariate analysis.

Most documents, including our previous study on a small cohort, didn’t demonstrate correlation between CD34 expression and GIST patients’ prognosis [49–51]. However, univariate analysis of present study revealed that CD34 positive GIST patients had better outcome than CD34 negative patients (5-yaer OS, 87.2% vs. 75.4%, P = 0.006; 5-year RFS, 80.6% vs. 64.3%, P = 0.001). Yet subsequent multivariate analysis didn’t show relationship between CD34 and patients’ survival. Therefore, further studies are required to determine the exact impact of sex and CD34 on GIST prognosis.

Long-term monitoring has shown that surgery alone is usually insufficient to control high-risk diseases. Introduction of imatinib has greatly improved the outcome of GIST. In China, the application of IM as adjuvant therapy was widely accepted not earlier than 2005. Presently, IM is standard therapy for advanced and primary intermediate-high risk GISTs (for adjuvant option) [14]. In present study, IM adjuvant therapy had better 5-year OS and RFS than non-adjuvant group. The limitation of this study is obvious: the selection of candidates and the interval of adjuvant therapy were not standardized. The follow-up period of adjuvant group was much shorter than that of non-adjuvant group, which highlighted the importance of persistent follow-up on those patients. The exact effect of IM on GIST can only be assessed by prospective randomized controlled trials with long-term follow-up, just like Z9001 [52] and SSGXVIII trials [53]. However, our findings still encourage the use of IM adjuvant therapy.

Undoubtedly, residual, recurrent or metastatic GISTs should be treated with imatinib according to the guidelines by European Society for Medical Oncology (ESMO) [54] or National Comprehensive Cancer Network (NCCN) [55]. In this cohort, however, some patients with advanced disease did not undergo IM therapy. Most of the cases were before 2005, when IM was not available in China. In present study, late-stage GIST (residual disease) patients underwent IM therapy had better 3-year OS and 1-year PFS than those who didn’t (75.6% vs. 6.8%; 87.6% vs. 12.4%, respectively), confirming the effect of IM on advanced disease.

## Conclusions

In summary, radical surgery is the treatment of choice for operable GISTs. Very low- and low-risk diseases can be treated with surgery alone. Lymph node metastasis is rare in GIST patients and may not be associated with poor prognosis. Large size, high mitotic rate, high risk group, and adjacent organ involvement all contribute to bad outcome of GISTs. IM therapy significantly improves survival of patients with intermediate-high risk or advanced GISTs.

## Electronic supplementary material

Additional file 1: **Univariate analysis of OS.** Univariate analysis of overall survival in 401 GIST patients (a: gender; b: tumor size; c: mitotic rate; d: CD34 expression; e: adjacent involvement). (PDF 160 KB)

Additional file 2: **Univariate analysis of RFS.** Univariate analysis of relapse-free survival in 401 GIST patients (a: sex; tumor site; c: tumor size; d: mitotic rate; e: CD34 expression; f: adjacent involvement). (PDF 191 KB)

Additional file 3: **Intermediate-high risk GIST.** Clinicopathological characteristics of 275 intermediate-high risk GIST patients according to whether received post-operation IM adjuvant therapy. (PDF 73 KB)

## Abbreviations

GIST: Gastrointestinal stromal tumor
NIH: National Institutes of Health
IM: Imatinib mesylate
OS: Overall survival
RFS: Relapse-free survival
GI: Gastrointestinal
ICC: Interstitial Cajal cell
PDGFRA: Platelet-derived growth factor receptor alpha
TKI: Tyrosine kinase inhibitor
HPF: High power field
IHCA: Immunohistochemistry assay
SMA: Smooth muscle actin
DOG1: (Discovered On GIST 1)
PFS: Progression-free survival
OR: Odds ratio
CI: Confidence interval
EGIST: Extragastrointestinal stromal tumor
IHC: Immunohistochemical
LNM: Lymph node metastasis
ESMO: European society for medical oncology
NCCN: National comprehensive cancer network.
