# Supplementary material for: Gastrointestinal stromal tumor: 15-years’ experience in a single center
Source: BMC Surg. 2014 Nov 18;14:93. doi: 10.1186/1471-2482-14-93 (PMC4254179; doi:10.1186/1471-2482-14-93)
Supplement: Supplementary file 1 — Additional file 1: Univariate analysis of OS. Univariate analysis of overall survival in 401 GIST patients (a: gender; b: tumor size; c: mitotic rate; d: CD34 expression; e: adjacent involvement). (PDF 160 KB) [file 12893_2014_534_MOESM1_ESM.pdf]

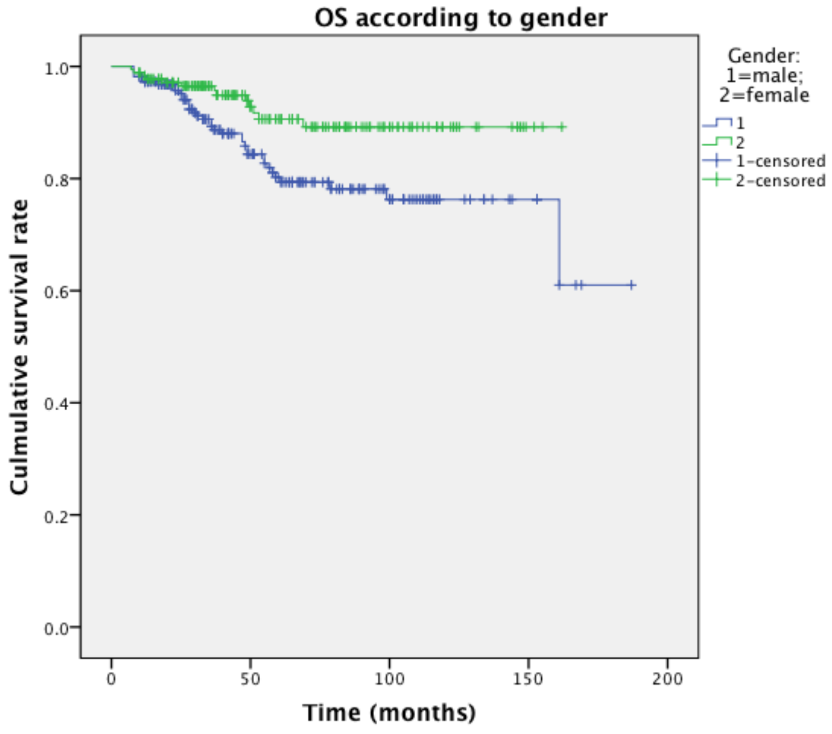
OS according to gender
Gender:
1=male;
2=female
1
2
1-censored
2-censored
Culmulative survival rate
1.0
0.8
0.6
0.4
0.2
0.0
0
50
100
150
200
Time (months)

a

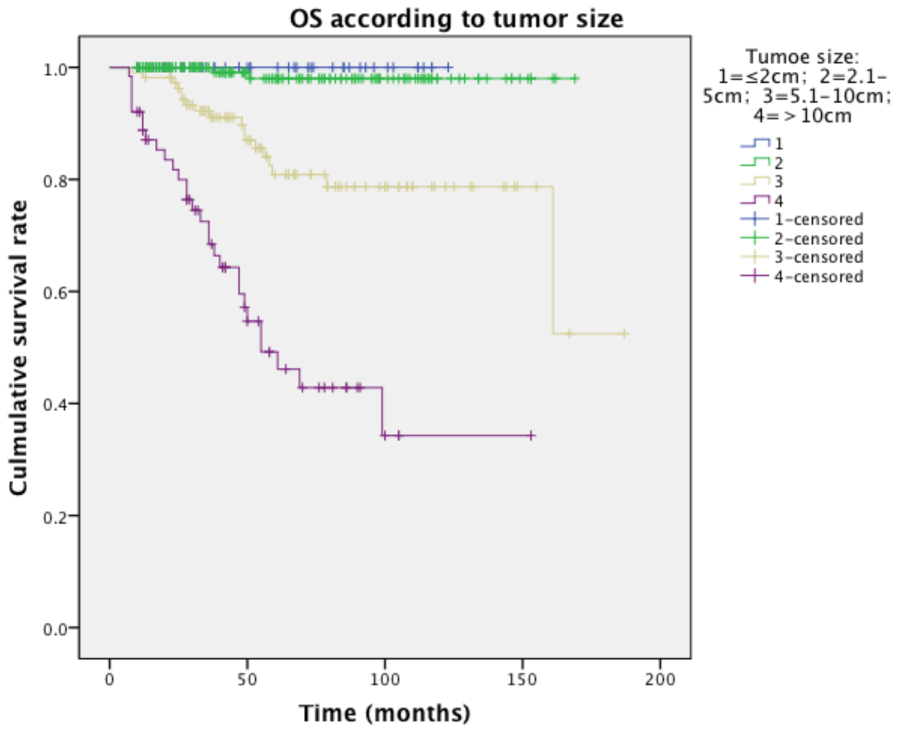
OS according to tumor size
Tumoe size:
1=≤2cm; 2=2.1-5cm; 3=5.1-10cm; 4=>10cm
1
2
3
4
1-censored
2-censored
3-censored
4-censored
Culmulative survival rate
1.0
0.8
0.6
0.4
0.2
0.0
0
50
100
150
200
Time (months)

b

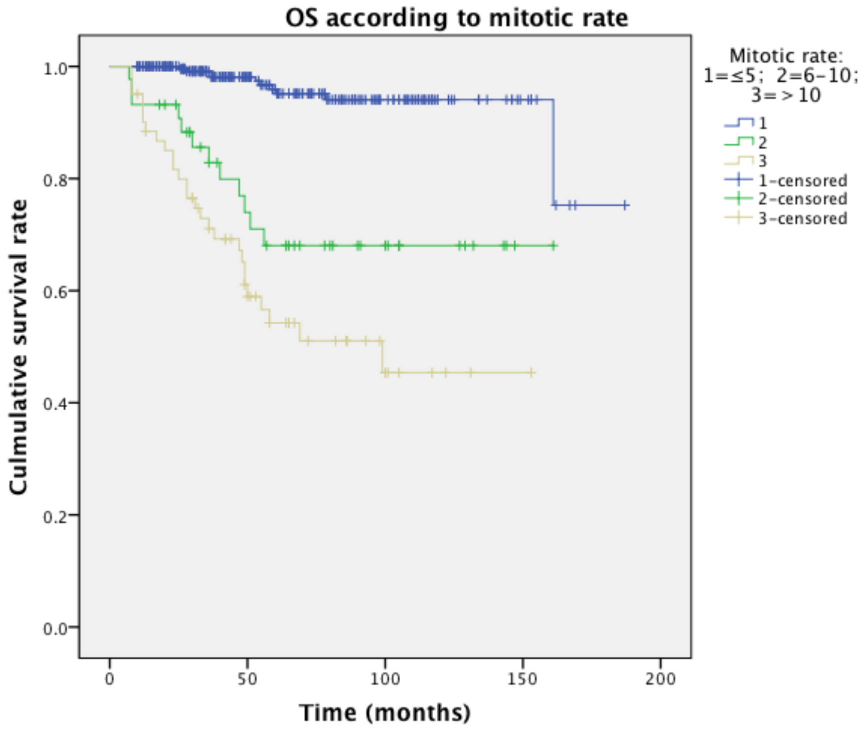
OS according to mitotic rate
Mitotic rate:
1=≤5; 2=6–10;
3=>10
1
2
3
1-censored
2-censored
3-censored
Culmulative survival rate
1.0
0.8
0.6
0.4
0.2
0.0
0
50
100
150
200
Time (months)

c

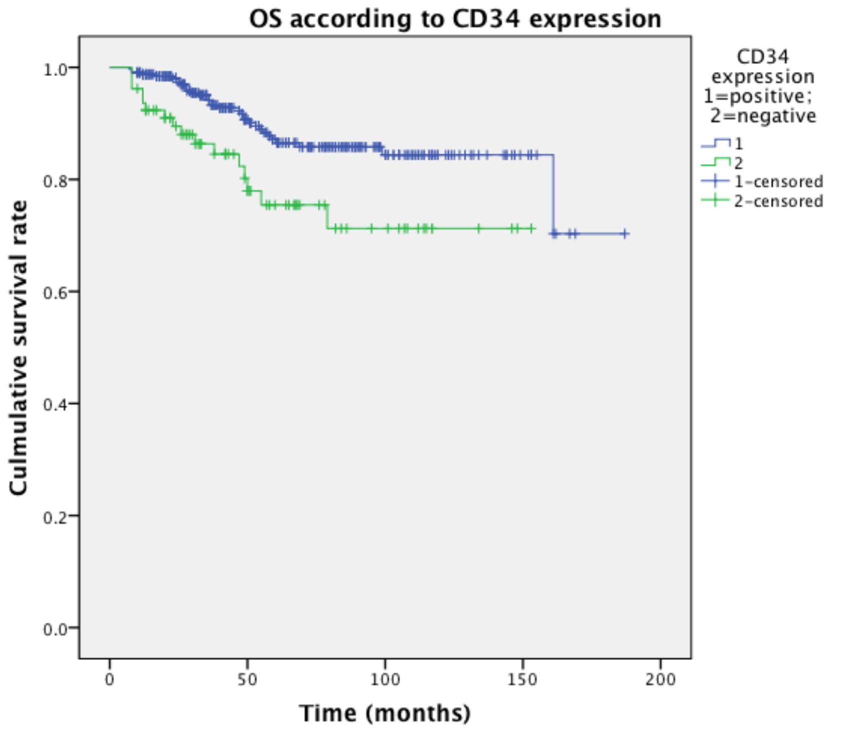
OS according to CD34 expression
CD34
expression
1=positive;
2=negative
1
2
1-censored
2-censored
Culmulative survival rate
1.0
0.8
0.6
0.4
0.2
0.0
0
50
100
150
200
Time (months)

d

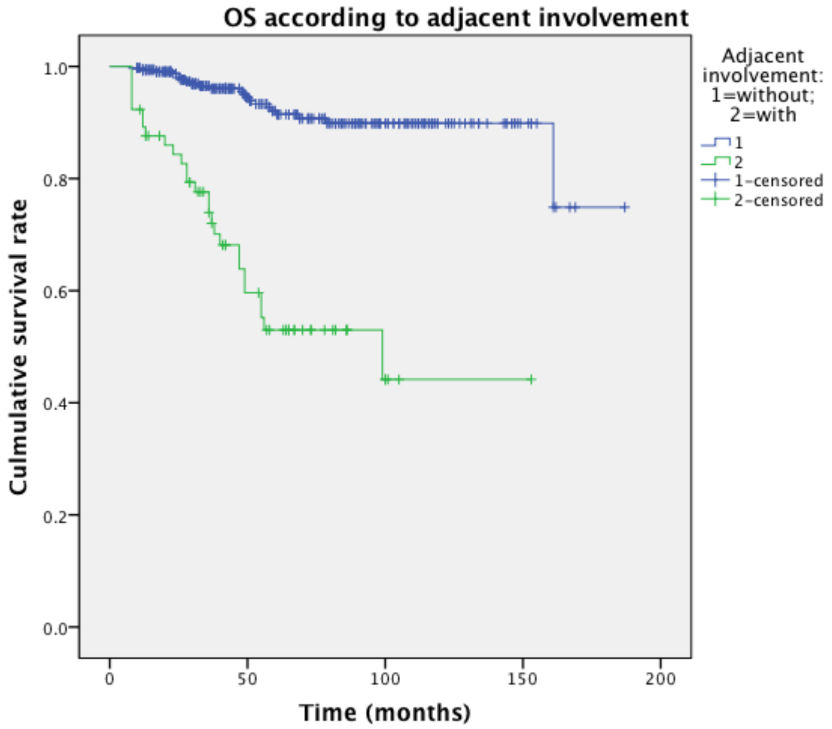

e

Additional file 1. Univariate analysis of overall survival in 401 GIST patients (a: gender; b: tumor size; c: mitotic rate; d: CD34 expression; e: adjacent involvement)
