# Supplementary material for: Gastrointestinal stromal tumor: 15-years’ experience in a single center
Source: BMC Surg. 2014 Nov 18;14:93. doi: 10.1186/1471-2482-14-93 (PMC4254179; doi:10.1186/1471-2482-14-93)
Supplement: Supplementary file 2 — Additional file 2: Univariate analysis of RFS. Univariate analysis of relapse-free survival in 401 GIST patients (a: sex; tumor site; c: tumor size; d: mitotic rate; e: CD34 expression; f: adjacent involvement). (PDF 191 KB) [file 12893_2014_534_MOESM2_ESM.pdf]

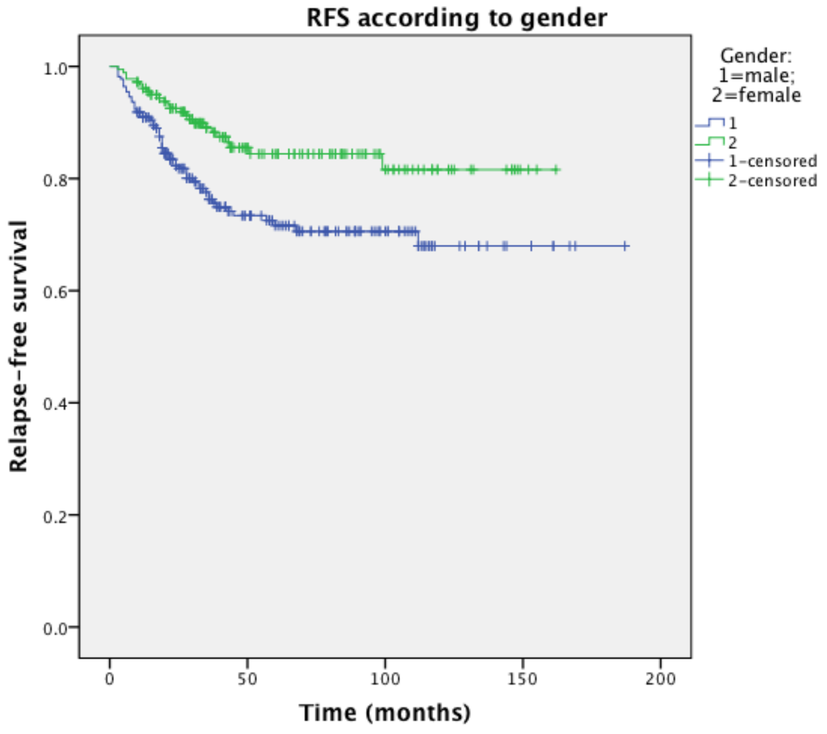
RFS according to gender
Gender:
1=male;
2=female
1
2
1-censored
2-censored
Relapse-free survival
1.0
0.8
0.6
0.4
0.2
0.0
0
50
100
150
200
Time (months)

a

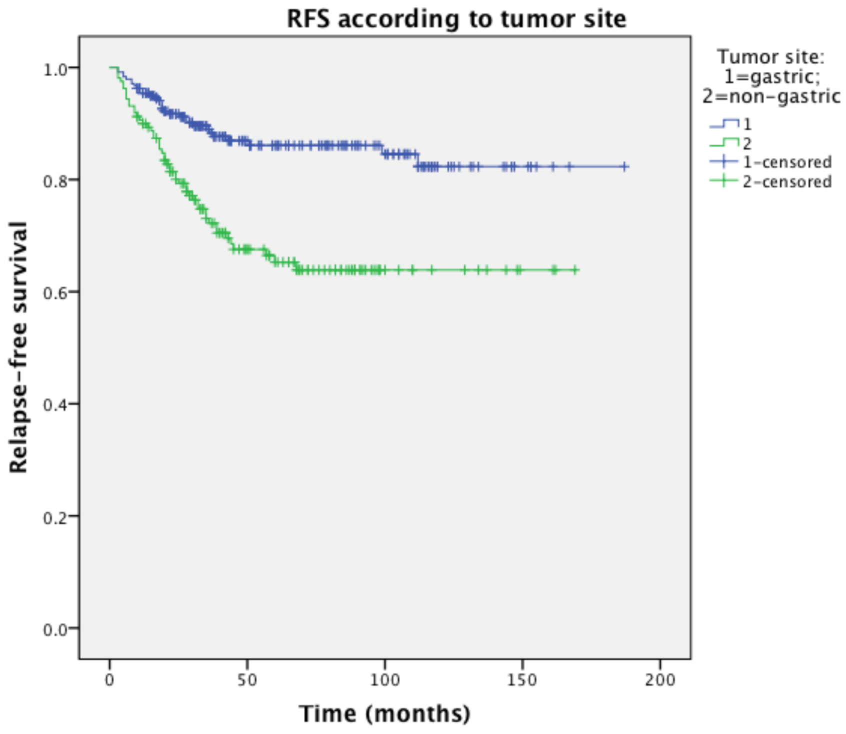
RFS according to tumor site
Tumor site:
1=gastric;
2=non-gastric
1
2
1-censored
2-censored
Relapse-free survival
1.0
0.8
0.6
0.4
0.2
0.0
0
50
100
150
200
Time (months)

b

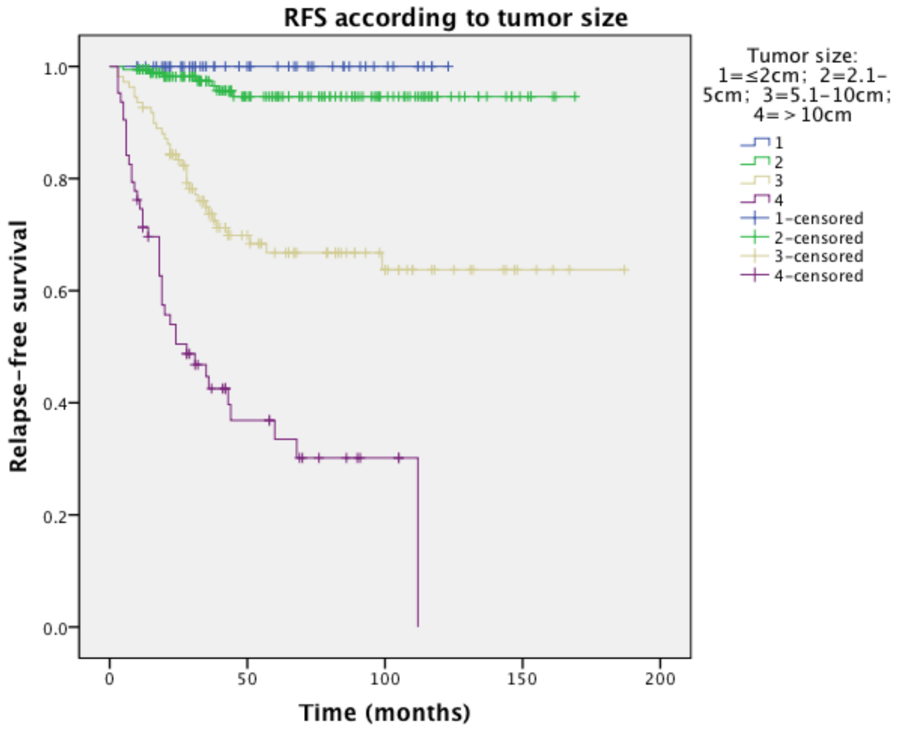
RFS according to tumor size
Relapse-free survival
Time (months)
Tumor size:
1=≤2cm; 2=2.1-5cm; 3=5.1-10cm; 4=>10cm
1
2
3
4
1-censored
2-censored
3-censored
4-censored
0.0
0.2
0.4
0.6
0.8
1.0
0
50
100
150
200

c

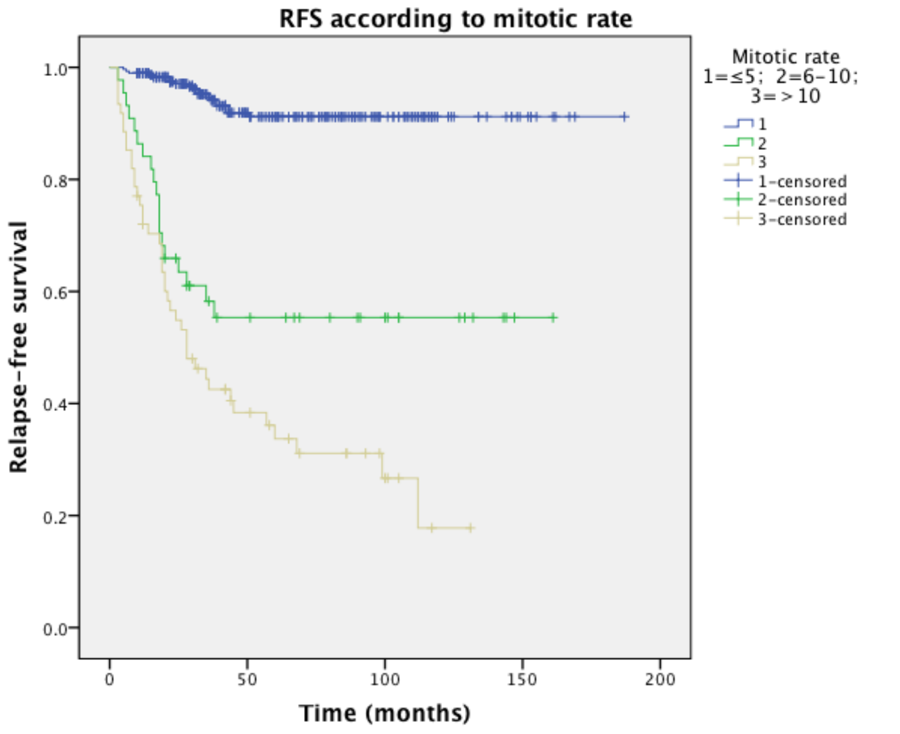
RFS according to mitotic rate
Relapse-free survival
Time (months)
Mitotic rate
1=≤5; 2=6-10; 3=>10
1
2
3
1-censored
2-censored
3-censored
0.0
0.2
0.4
0.6
0.8
1.0
0
50
100
150
200

d

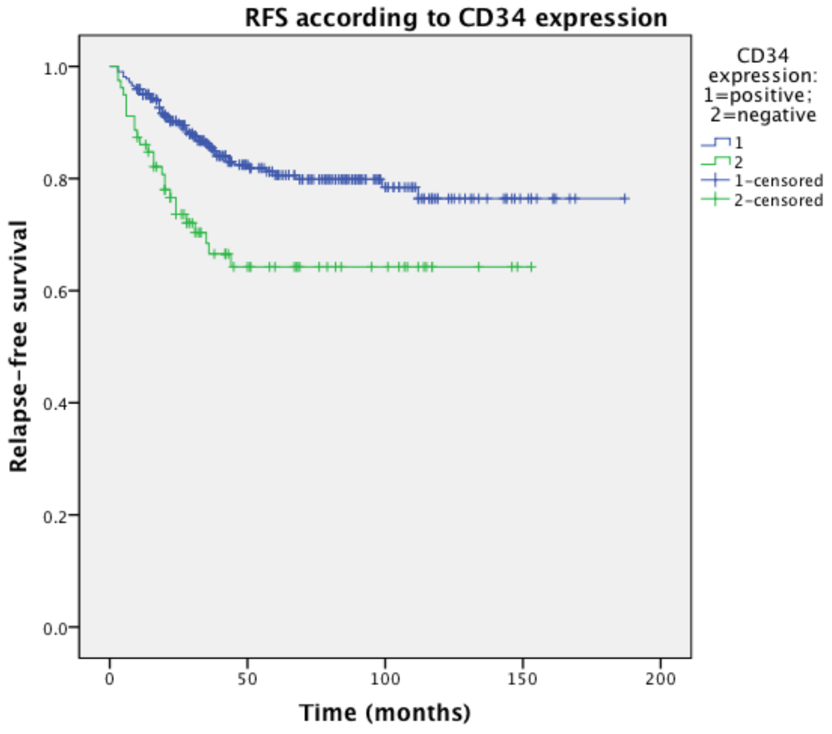

e

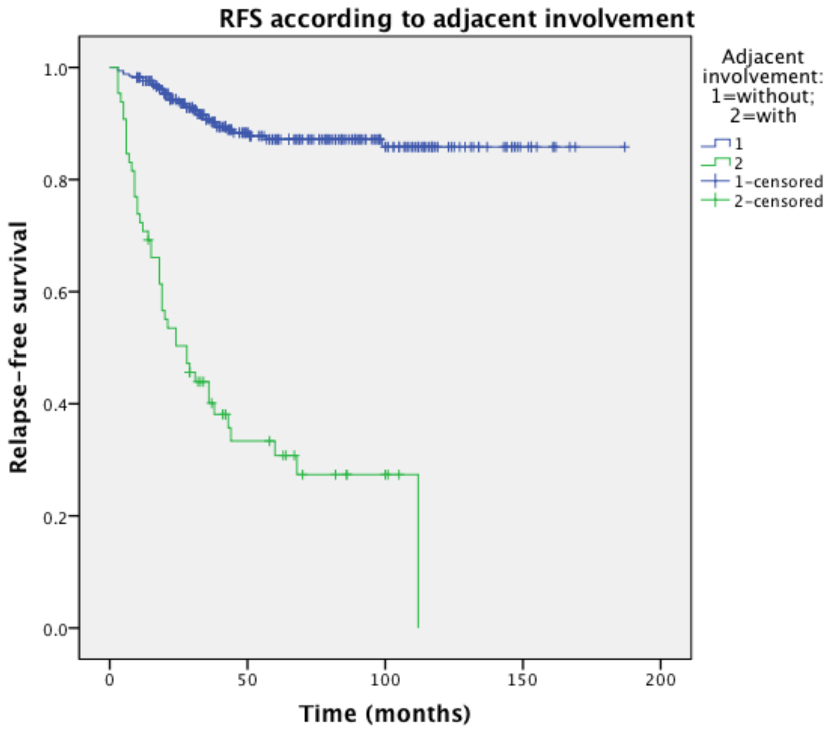

f

Additional file 2. Univariate analysis of relapse-free survival in 401 GIST patients (a: sex; tumor site; c: tumor size; d: mitotic rate; e: CD34 expression; f: adjacent involvement)
