# Supplementary material for: Gastrointestinal stromal tumor: 15-years’ experience in a single center
Source: BMC Surg. 2014 Nov 18;14:93. doi: 10.1186/1471-2482-14-93 (PMC4254179; doi:10.1186/1471-2482-14-93)
Supplement: Supplementary file 3 — Additional file 3: Intermediate-high risk GIST. Clinicopathological characteristics of 275 intermediate-high risk GIST patients according to whether received post-operation IM adjuvant therapy. (PDF 73 KB) [file 12893_2014_534_MOESM3_ESM.pdf]

Additional file 3. Clinicopathological characteristics of 275 intermediate-high risk GIST patients according to whether received post-operation IM adjuvant therapy

Feature	Group	N	Non-adjuvant	Adjuvant	χ^2	P-value
Gender	Male	170	121	49	1.629	0.230
	Female	105	67	38		
Age	≤60	129	88	41	0.002	1.000
	＞60	146	100	46		
Site	Gastric	143	93	50	1.526	0.244
	Non-gastric	132	95	37		
Size	≤2cm	1	1	0	1.972	0.578
	2.1-5cm	24	19	5		
	5.1-10cm	159	106	53		
	＞10cm	91	62	29		
Mitotic rate	＜5/50HPF	131	85	46	1.452	0.484
	5~10/50HPF	61	43	18		
	＞10/50HPF	83	60	23		
Risk class	Intermediate risk	79	51	28	0.743	0.394
	High risk	196	137	59		
CD117 expression	Positive	242	166	76	0.050	0.843
	Negative	33	22	11		
CD34 expression	Positive	213	143	70	0.658	0.443
	Negative	62	45	17		
Adjacent involvement	Without	184	126	58	0.003	1.000
	With	91	62	29		
